# Association of Left Ventricular Mass with All-Cause Mortality, Myocardial Infarction and Stroke

**DOI:** 10.1371/journal.pone.0045570

**Published:** 2012-09-26

**Authors:** Alberto Bouzas-Mosquera, Francisco J. Broullón, Nemesio Álvarez-García, Jesús Peteiro, Víctor X. Mosquera, Alfonso Castro-Beiras

**Affiliations:** 1 Department of Cardiology, Hospital Universitario A Coruña, A Coruña, Spain; 2 Spanish Cooperative Cardiovascular Disease Research Network (RECAVA) - Instituto de Salud Carlos III, Madrid, Spain; 3 Department of Information Technology, Hospital Universitario A Coruña, A Coruña, Spain; 4 Department of Cardiac Surgery, Hospital Universitario A Coruña, A Coruña, Spain; College of Pharmacy, University of Florida, United States of America

## Abstract

**Background:**

Our aim was to assess the association of left ventricular mass with mortality and nonfatal cardiovascular events.

**Methodology/Principal Findings:**

Left ventricular mass was measured by echocardiography in 40138 adult patients (mean age 61.1±16.4 years, 52.5% male). The primary endpoint was all-cause mortality. Secondary endpoints included nonfatal myocardial infarction and nonfatal stroke. During a mean follow-up period of 5.6±3.9 years, 9181 patients died, 901 patients had a nonfatal myocardial infarction, and 2139 patients had a nonfatal stroke. Cumulative 10-year mortality was 26.8%, 31.9%, 37.4% and 46.4% in patients with normal, mildly, moderately and severely increased left ventricular mass, respectively (p<0.001). Ten-year rates of nonfatal myocardial infarction and stroke ranged from 3.2% and 6.7% in patients with normal left ventricular mass to 5.3% and 12.7% in those with severe increase in left ventricular mass, respectively. After multivariate adjustment, left ventricular mass remained an independent predictor of all-cause mortality (hazard ratio [HR] per 100 g increase 1.21, 95% confidence interval [CI] 1.14–1–27, p<0.001 in women, and HR 1.09, 95% CI 1.04–1–13, p<0.001 in men), myocardial infarction (HR 1.60, 95% CI 1.31–1.94, p<0.001 in women and HR 1.15, 95% CI 1.02–1.29, p = 0.019 in men) and stroke (HR 1.26, 95% CI 1.13–1.40, p<0.001 in women and HR 1.19, 95% CI 1.09–1.30, p<0.001 in men).

**Conclusions/Significance:**

Left ventricular mass has a graded and independent association with all-cause mortality, myocardial infarction and stroke.

## Introduction

Left ventricular hypertrophy may be a consequence of the effect of a chronically increased left ventricular afterload, such as in systemic hypertension or aortic stenosis, or a result of an intrinsic abnormality of myocites. Echocardiography allows an estimation of left ventricular mass, and has shown to be more sensitive for the detection of left ventricular hypertrophy than electrocardiography. Among other detrimental effects, left ventricular hypertrophy may lead to diastolic dysfunction, may reduce coronary flow reserve and may facilitate ventricular arrhythmias [1]. Thus, echocardiographically determined left ventricular mass may provide important prognostic information [2]–[5]. Our aim was to assess the association of left ventricular mass with all-cause mortality, myocardial infarction and stroke in a large cohort of adult patients referred for echocardiography.

## Methods

### Ethics Statement

This study was approved by the Comité Ético de Investigación Clínica de Galicia (our local research ethics committee), which waived the requirement for patient consent.

### Study Cohort

A total of 66786 comprehensive transthoracic echocardiographic studies (including determination of left ventricular mass) performed at our unit in a sample of community-based patients between January 1, 1994 and March 11, 2008 were initially considered for inclusion in this study. Patients were excluded if they were <18 years or had previously undergone cardiac transplantation. In case of repeated studies, the first one was selected. A total of 652 patients for whom no follow-up data were available were also excluded. Thus, the final study population was made up of 40138 patients.

### Data Sources and Study Covariates

Demographic, clinical and echocardiographic data were entered in a dedicated database at the time of the original echocardiograms. The study was completed using linked clinical and administrative databases through the Department of Information Technology of our institution. Data sources included discharge code and laboratory databases, as well as other clinical databases from several units of our department and from the department of Cardiac Surgery. These data sources were linked using unique identifiers.

Echocardiograms were performed in the left lateral decubitus position using standard imaging planes according to the American Society of Echocardiography (ASE) recommendations [6]. Left ventricular end-diastolic diameter, ventricular septal thickness and posterior wall thickness were measured by M-mode or 2-dimensional echocardiography in end-diastole (ie, in the frame in the cardiac cycle in which the left ventricular dimension was largest). The left ventricular mass was calculated using the formula by Devereux et al [7]:

*Left ventricular mass (g) = 0.80×{1.04×[(ventricular septal thickness+left ventricular end-diastolic diameter+posterior wall thickness)^3^−(left ventricular end-diastolic diameter)^3^]} +0,6 g.*

Left ventricular mass was categorized as a sex-specific variable. Left ventricular hypertrophy was defined as a left ventricular mass ≥163 g in women or ≥224 g in men [8]. A left ventricular mass below these values was considered normal. Left ventricular hypertrophy was further classified as mild (163–186 g in women or 225–258 g in men), moderate (187–210 g in women or 259–292 g in men) and severe (≥211 g in women or ≥293 g in men) in accordance with the ASE recommendations [8]. Left ventricular ejection fraction was estimated by the Teichholz’s formula or the Simpson’s rule. Left ventricular systolic dysfunction was defined as left ventricular ejection fraction <55% [8]. Left ventricular enlargement was defined as an end-diastolic left ventricular diameter ≥60 mm in men or ≥54 mm in women [8]. Left atrial diameter was measured from the posterior aortic wall to the posterior left atrial wall in the parasternal long-axis view at the end-ventricular systole. The presence and severity of aortic stenosis was determined by measuring peak and mean gradients across the aortic valve by continuous-wave Doppler or by calculating the aortic valve area using the continuity equation [9]. The degree of mitral regurgitation was determined semiquantitatively based on parameters such as regurgitant jet area, jet profile in continuous-wave Doppler, proximal isovelocity surface area, vena contracta, and pulsed Doppler quantitative flow methods [10].

Blood pressure was not routinely measured at the time of the echocardiograms, and the definition of hypertension was mainly based on previously established diagnoses. Diabetes mellitus was defined as fasting serum glucose ≥126 mg/dL or nonfasting glucose of ≥200 mg/dL in any prior laboratory test. Hypercholesterolemia was defined as total cholesterol ≥220 mg/dL. A history of atrial fibrillation was defined as atrial fibrillation recorded at the time of the echocardiograms or any previously known episode of atrial fibrillation. Chronic kidney disease was defined as an estimated glomerular filtration rate <60 mL/minute/1.73 m2 by the Modification of Diet in Renal Disease Study equation in at least 2 prior observations more than 90 days apart. Chronic obstructive pulmonary disease was defined as International Classification of Diseases, Ninth Revision (ICD-9) codes 491.×, 492.×, and 496.

### Study Outcomes

Follow-up data were retrieved from healthcare databases, electronic medical records and death certificates. The primary endpoint was all-cause mortality. Secondary endpoints included nonfatal myocardial infarction and nonfatal stroke. Increased CK-MB and/or troponin I values during follow-up were retrieved from comprehensive laboratory databases and matched with ICD-9 discharge code databases (code 410) for the possible diagnosis of myocardial infarction; electronic medical records were reviewed in case of discrepancy. Stroke during follow-up was ascertained by the review of ICD-9 discharge codes, and classified as hemorrhagic (codes 430–432) or ischemic (codes 433.×1, 434.×1, and 436). Patients were censored at the last known alive date as of April 2009. Those who underwent cardiac transplantation during follow-up (n = 246) were censored at the time of the procedure. Mean follow-up was 5.6±4 years (interquartile range 2.4–8.8 years).

### Statistical Analysis

Categorical variables were reported as percentages and comparison between groups based on the χ^2^ test (or the χ^2^ test for the trend). Continuous variables were reported as mean ± standard deviation and differences were assessed using the Student t, the one-way ANOVA, the Mann-Whitney U or the Kruskal-Wallis tests as appropriate. Cumulative event curves were estimated by the Kaplan-Meier method and compared by the log-rank test, with left ventricular mass stratified according to the sex specific ASE criteria. Cox proportional hazards regression models were used to assess the associations of left ventricular mass with the endpoints. Hazard ratios (HR) with 95% confidence intervals (CI) were estimated. In these analyses, left ventricular mass was included as a continuous variable and stratified according to sex. The assumption of proportionality of hazards was verified using log-minus-log survival plots. Multivariate analyses were adjusted for the following covariates: age, gender, hypertension, diabetes mellitus, hypercholesterolemia, tobacco use, previous stroke or transient ischemic attack, history of atrial fibrillation, history of congestive heart failure, prior myocardial infarction, history of coronary revascularization, left ventricular ejection fraction, left atrial diameter, severity of aortic stenosis, severity of mitral regurgitation, history of cancer, chronic kidney disease, chronic obstructive pulmonary disease and anticoagulant therapy. The independent association of left ventricular mass with mortality, myocardial infarction and stroke was also verified among subgroups according to age (< or ≥65 years), hypertension and left ventricular ejection fraction (< or ≥55%). Statistical analyses were performed using Statistical Package for the Social Sciences software, version 15.0 (SPSS, Chicago, Illinois).

## Results

### Baseline Characteristics

Patients had a mean age of 61.1±16.4 years, and 21068 (52.5%) were male. Mean left ventricular mass was 204±80 g (177±66 g in women and 228±20 g in men). Left ventricular hypertrophy was present in 19104 patients (47.6%), of which 10023 (52.5%) were women and 9081 (47.5%) were men. According to the ASE criteria, left ventricular mass was normal in 21034 patients (52.4%), mildly increased in 6429 patients (16%), moderately increased in 4365 patients (10.9%), and severely increased in 8310 patients (20.7%). Table 1 compares the demographic, clinical and echocardiographic variables according to the degree of increase in left ventricular mass. Patients with left ventricular hypertrophy were older and were more likely to have a history of hypertension, atrial fibrillation, diabetes mellitus, congestive heart failure, left ventricular systolic dysfunction, significant aortic stenosis and chronic kidney disease.

**Table 1:** Baseline characteristics of the patients according to the degree of increase in left ventricular mass.

	Degree of increase in left ventricular mass				p*
	Normal	Mild	Moderate	Severe	
Male sex, No (%)	11987 (43.0)	3340 (52.0)	2071 (47.4)	3670 (44.2)	<0.001
Age, mean (SD), y	57.2±17.9	63.6±14.4	65.4±13.3	66.8±12.4	<0.001
Hypertension, No (%)	8167 (38.8)	3452 (53.7)	2638 (60.4)	5428 (65.3)	<0.001
Diabetes mellitus, No (%)	4459 (21.2)	1598 (24.9)	1176 (26.9)	2511 (30.2)	<0.001
Hypercholesterolemia, No (%)	9233 (43.9)	2985 (46.4)	2014 (46.1)	3630 (43.7)	0.6
Smoking, No (%)	5228 (24.9)	1567 (24.4)	1025 (23.5)	1928 (23.2)	<0.001
History of atrial fibrillation, No (%)	3586 (17.0)	1533 (23.8)	1124 (25.8)	2292 (27.6)	<0.001
History of stroke/TIA, No (%)	2025 (9.6)	565 (8.8)	390 (8.9)	605 (7.3)	<0.001
Prior myocardial infarction, No (%)	1647 (7.8)	641 (10.0)	465 (10.7)	847 (10.2)	<0.001
Coronary revascularization, No (%)	929 (4.4)	337 (5.2)	198 (4.5)	350 (4.2)	0.57
PCI, No (%)	649 (3.1)	204 (3.2)	121 (2.8)	200 (2.4)	0.002
CABG, No (%)	315 (1.5)	144 (2.2)	84 (1.9)	163 (2.0)	0.003
History of congestive heart failure, No (%)	1502 (7.1)	798 (12.4)	667 (15.3)	1857 (22.3)	<0.001
Valve prosthesis, No (%)	219 (1.0)	112 (1.7)	111 (2.5)	266 (3.2)	<0.001
History of cancer, No (%)	1492 (7.1)	414 (6.4)	244 (5.6)	419 (5.0)	<0.001
Chronic kidney disease, No (%)	4622 (22.0)	1699 (26.4)	1292 (29.6)	2653 (31.9)	<0.001
COPD, No (%)	1088 (5.2)	330 (5.1)	199 (4.6)	478 (5.8)	0.20
Oral anticoagulant therapy, No (%)	1143 (5.4)	444 (6.9)	321 (7.4)	628 (7.6)	<0.001
LVEF, mean (SD), %	66.6±10.9	64.8±13.0	63.6±14.1	59.7±16.4	<0.001
End-diastolic LV diameter, mean (SD), mm	45.1±5.7	48.7±6.1	50.2±6.6	54.0±8.9	<0.001
End-systolic LV diameter, mean (SD), mm	28.3±5.9	31.1±7.3	32.5±8.2	36.5±11.1	<0.001
LV enlargement, No (%)	258 (1.2)	387 (6.0)	520 (11.9)	2773 (33.4)	<0.001
Left atrial diameter, mean (SD), mm	37.8±6.6	41.2±6.9	42.4±6.9	45.1±7.9	<0.001
Moderate or severe AS, No (%)	709 (3.4)	403 (6.3)	359 (8.2)	1231 (14.8)	<0.001
Moderate or severe MR, No (%)	544 (2.6)	347 (5.4)	240 (5.5)	838 (10.1)	<0.001

AS = aortic stenosis; CABG = coronary artery bypass grafting; COPD = chronic obstructive pulmonary disease; LV = left ventricular; LVEF = LV ejection fraction; MR = mitral regurgitation; PCI = percutaneous coronary intervention; TIA = transient ischemic attack.

* P value for the trend.

### Left Ventricular Mass and Mortality

**Figure 1:**
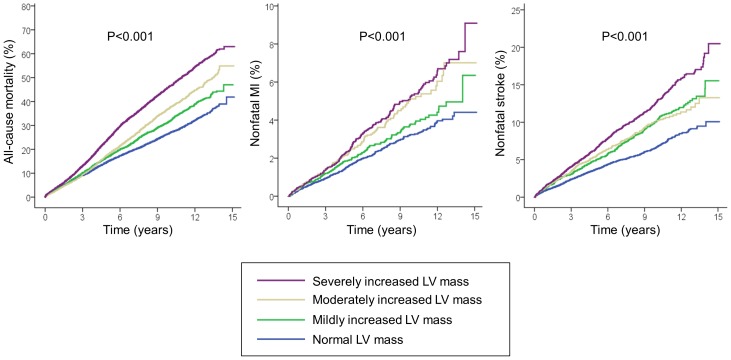
Kaplan-Meier curves for all-cause mortality, nonfatal myocardial infarction and stroke stratified according to left ventricular mass.

**Figure 2:**
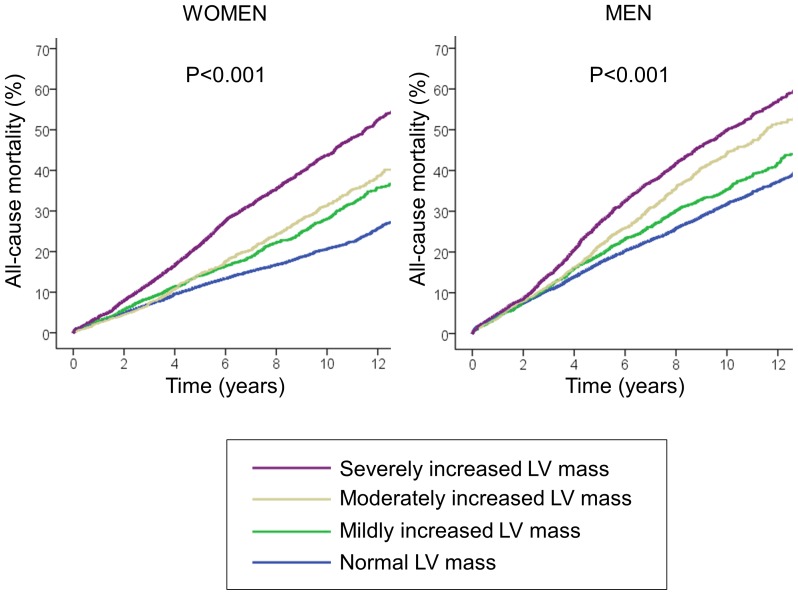
Kaplan-Meier curves for all-cause mortality according to left ventricular mass stratified by sex.

**Figure 3:**
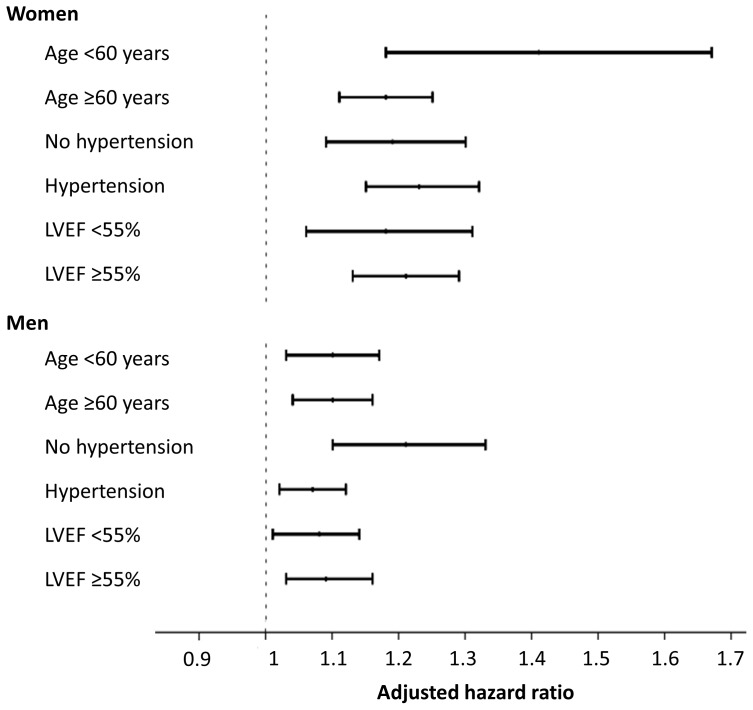
Hazard ratios for all-cause mortality per 100 g increase in left ventricular mass according to sex and stratified by age, hypertension and left ventricular ejection fraction (LVEF).

During follow-up, a total of 9181 patients died. Crude cumulative 10-year mortality was 26.8% in patients with normal left ventricular mass, 31.9% in those with mild increase in left ventricular mass, 37.4% in those with moderately increased left ventricular mass and 46.4% in those with severe increase in left ventricular mass (p<0.001, Figure 1). This graded association was consistent among both sexes (Figure 2). The unadjusted hazard ratio [HR] for mortality per 100 g increase in left ventricular mass was 1.56 for women (95% confidence interval [CI] 1.50–1.62, p<0.001), and 1.26 for men (95% CI 1.22–1.29, p<0.001). After adjustment in multivariable Cox proportional hazard analysis, left ventricular mass remained an independent predictor of all-cause mortality in both sexes (Table 2). These results did not change significantly after excluding patients with moderate or severe aortic stenosis (HR per 100 g increase 1.24, 95% CI 1.17–1.32, p<0.001 for women, and HR 1.11, 95% CI 1.07–1.16, p<0.001 for men). Figure 3 shows the adjusted HR and 95% CI of left ventricular mass for predicting mortality stratified by sex and a range of clinically-relevant subgroups.

### Left Ventricular Mass and Nonfatal Cardiovascular Events

**Table 2:** Multivariable adjusted hazard ratios per 100 g increase in left ventricular mass (95% confidence intervals) for all-cause mortality, nonfatal myocardial infarction and nonfatal stroke in women and men.

	Women (N = 19070)			Men (N = 21068)		
	Number of events	Adjusted HR (95% CI)*	p	Number of events	Adjusted HR (95% CI)*	p
All-cause mortality	3020	1.21 (1.14–1.27)	<0.001	5261	1.09 (1.04–1.13)	<0.001
Nonfatal MI	254	1.60 (1.31–1.94)	<0.001	647	1.15 (1–02–1.29)	0.019
Any nonfatal stroke	1022	1.26 (1.13–1.40)	<0.001	1117	1.19 (1.09–1.30)	<0.001
Nonfatal ischemic stroke	885	1.24 (1.10–1.39)	<0.001	923	1.18 (1.07–1.30)	<0.001
Nonfatal hemorrhagic stroke	169	1.26 (0.97–1.65)	0.09	231	1.23 (1.02–1.48)	0.03

CI = confidence interval; HR = hazard ratio; MI = myocardial infarction.

* Adjusted by age, gender, tobacco use, hypertension, diabetes mellitus, hypercholesterolemia, tobacco use, previous stroke or transient ischemic attack, history of atrial fibrillation, history of congestive heart failure, prior myocardial infarction, history of coronary revascularization, left ventricular ejection fraction, left atrial diameter, severity of aortic stenosis, severity of mitral regurgitation, history of cancer, chronic kidney disease, chronic obstructive pulmonary disease and anticoagulant therapy.

Overall, 901 patients had a nonfatal myocardial infarction, 1808 patients had an ischemic stroke, 400 patients had a hemorrhagic stroke, and 2139 patients had at least one nonfatal stroke during follow-up. As compared with patients with normal left ventricular mass, those with left ventricular hypertrophy had a higher rate of nonfatal cardiovascular events (Figure 1). Ten-year rates of nonfatal myocardial infarction and stroke ranged from 3.2% and 6.7% in patients with normal left ventricular mass to 5.3% and 12.7% in those with severe increase in left ventricular mass, respectively. The unadjusted hazard ratio for nonfatal myocardial infarction was 1.70 per 100 g increase in left ventricular mass in women (95% CI 1.48–1.95, p<0.001), and 1.22 in men (95% CI 1.12–1.32, p<0.001), while HR for nonfatal stroke was 1.56 in women (95% CI 1.45–1.67, p<0.001) and 1.24 in men (95% CI 1.16–1.32, p<0.001). After multivariate adjustment, left ventricular mass also remained independently and significantly associated with nonfatal myocardial infarction and stroke (Table 2). The exclusion of patients with significant aortic stenosis did not modify these results either (adjusted HR for nonfatal myocardial infarction per 100 g increase 1.57 in women, 95% CI 1.26–1.95, p<0.001, and HR 1.17 in men, 95% CI 1.03–1.32, p<0.001; HR for nonfatal stroke 1.28 in women, 95% CI 1.14–1.44, p<0.001, and HR 1.21 in men, 95% CI 1.10–1.32, p<0.001). When the specific types of stroke were analyzed separately, left ventricular mass was significantly associated with ischemic stroke in both sexes and with hemorrhagic stroke in men, with a nonsignificant trend in women (Table 2).

## Discussion

In a large sample of patients referred for transthoracic echocardiography, this study shows that left ventricular mass has an independent and graded association with all-cause mortality and nonfatal cardiovascular events. To our knowledge, this is the largest study assessing the value of echocardiographically determined left ventricular mass for predicting mortality, myocardial infarction and stroke.

Previous studies have found that left ventricular mass as assessed by echocardiography is associated with an increased risk of mortality and morbidity beyond the risk associated with hypertension alone. In a population of 3220 subjects included in the Framingham Heart Study [5], left ventricular mass was associated with the risk of cardiovascular disease, cardiovascular death and all-cause mortality, independently of traditional cardiovascular risk factors. The same group also reported a higher incidence of coronary heart disease and stroke in elderly subjects [2], [4]. In our study, left ventricular mass had a greater impact on outcome in women than it had in men; this is consistent with a prior study by Liao et al [11], who found that the risk of all-cause mortality and cardiac death attributable to left ventricular hypertrophy in a population of 436 black subjects was higher in women. However, although in our study left ventricular mass was significantly associated with ischemic stroke in both sexes, it was associated with hemorrhagic stroke only in men, with a nonsignificant trend in women, perhaps due to lack of statistical power.

Due to the large number of variables analyzed, we also stratified the results by those we considered more clinically relevant, such as age, hypertension and left ventricular ejection fraction. An increase in left ventricular mass in younger patients and in those without hypertension was associated with higher mortality rate that the same increase in older patients or in those with hypertension, although the 95% confidence intervals overlap, and this should be interpreted with caution. The population evaluated in our study was almost entirely Caucasian, although the value of left ventricular mass for predicting outcome has also been demonstrated in other ethnic populations, such as Hispanics [12] and African Americans [13], [14]. We assessed the value of left ventricular mass for predicting outcome in patients referred for clinical reasons. Our results expand on those of previous studies assessing the prognostic value of left ventricular mass in patients with essential hypertension [15], [16], coronary artery disease [17], [18] and chronic kidney disease [19], [20].

The mechanisms accounting for the association of left ventricular mass with outcome have not been fully unraveled. Left ventricular hypertrophy increases myocardial oxygen consumption, which may lead to an imbalance between the demand and the supply of oxygen to the myocardium and, thus, to myocardial ischemia [21].

Increased left ventricular mass also predisposes to ventricular arrhythmias and sudden death [3]. This susceptibility may be mediated by a prolongation of the action potential duration and refractoriness, and an impaired ability to handle intracellular calcium by the hypertrophied myocardium [22]. Coexisting myocardial ischemia and an increase in ventricular wall stress may also facilitate ventricular arrhythmias [22]. Left ventricular hypertrophy may also contribute to diastolic dysfunction, which may lead to heart failure. Finally, left ventricular hypertrophy may be also a marker of atherosclerosis and reflect a prolonged exposure to other cardiovascular risk factors, such as hypertension.

Our study has several limitations. First, this was an observational study and, as such, residual confounding may account for at least part of the observed differences in outcome. We did not account for body mass index, which in turn may be related to outcome. In addition, antihypertensive drugs were not available for analysis; some studies [23], [24] have found that left ventricular hypertrophy can be reversed by pharmacologic interventions, and this may lead to a reduction in cardiovascular events. Although the criteria for measuring left ventricular mass did not change over time, no quality control procedures to ensure comparability of left ventricular mass measurements over time were implemented, and we cannot rule out that changes in the echocardiographic equipment or differences in the assessment of left ventricular mass by varying cardiologists over time might have affected the results. Finally, there are a number of alternative echocardiographic methods that allow a more reliable estimation of left ventricular mass [8]. Cardiac magnetic resonance imaging is also more accurate for this purpose [25]; nonetheless, echocardiography is less expensive and more widely available.

In conclusion, left ventricular mass has a graded and independent association with all-cause mortality, myocardial infarction and stroke beyond that associated with other traditional risk factors in unselected patients referred for echocardiography. The degree of refinement of the prognostic value of left ventricular mass assessed by alternative echocardiographic methods and other noninvasive imaging techniques merits further investigation.
